# Non-aqueous, zwitterionic solvent as an alternative for dimethyl sulfoxide in the life sciences

**DOI:** 10.1038/s42004-020-00409-7

**Published:** 2020-11-11

**Authors:** Kosuke Kuroda, Tetsuo Komori, Kojiro Ishibashi, Takuya Uto, Isao Kobayashi, Riki Kadokawa, Yui Kato, Kazuaki Ninomiya, Kenji Takahashi, Eishu Hirata

**Affiliations:** 1 Faculty of Biological Science and Technology, Institute of Science and Engineering, Kanazawa University, Kakuma-machi, Kanazawa, 920-1192 Japan; 2 Division of Tumor Cell Biology and Bioimaging, Cancer Research Institute of Kanazawa University, Kakuma-machi, Kanazawa, 920-1192 Japan; 3 Organization for Promotion of Tenure Track, University of Miyazaki, Nishi 1-1 Gakuen-Kibanadai, Miyazaki, 889-2192 Japan; 4 Institute for Frontier Science Initiative, Kanazawa University, Kakuma-machi, Kanazawa, 920-1192 Japan; 5 Nano Life Science Institute of Kanazawa University, Kakuma-machi, Kanazawa, 920-1192 Japan

**Keywords:** Biochemistry, Chemical biology, Chemical safety, Ionic liquids

## Abstract

Dimethyl sulfoxide (DMSO) is widely used as a solvent in the life sciences, however, it is somewhat toxic and affects cell behaviours in a range of ways. Here, we propose a zwitterionic liquid (ZIL), a zwitterion-type ionic liquid containing histidine-like module, as a new alternative to DMSO. ZIL is not cell permeable, less toxic to cells and tissues, and has great potential as a vehicle for various hydrophobic drugs. Notably, ZIL can serve as a solvent for stock solutions of platinating agents, whose anticancer effects are completely abolished by dissolution in DMSO. Furthermore, ZIL possesses suitable affinity to the plasma membrane and acts as a cryoprotectant. Our results suggest that ZIL is a potent, multifunctional and biocompatible solvent that compensates for many shortcomings of DMSO.

## Abstract (web summary)

Dimethyl sulfoxide is a commonly used solvent, but its permeability and effect on cells is problematic. Here, an alternative solvent, a zwitterion-type ionic liquid, and its plasma membrane affinity as well as cryo-protecting property and drug-dissolution ability are reported.

## Introduction

Dimethyl sulfoxide (DMSO) is the most commonly used nonaqueous solvent and is classified as class 3 (nontoxic) by the United States Food and Drug Administration (FDA) and the International Council for Harmonisation of Technical Requirements for Pharmaceuticals for Human Use (ICH)^1,2^. Because DMSO serves not only as a vehicle for various hydrophobic compounds but also as a cryoprotectant for cell storage, most researchers and engineers in life sciences (hundreds of thousands to millions of people) use DMSO and it has been recognised as an irreplaceable solvent^3–7^. On the other hand, DMSO is somewhat toxic and affects cell behaviours in a range of ways. Mechanistically, DMSO is a cell-permeable reagent known to bind to various proteins in the cytoplasm or nucleus^8^, which directly (chemically and physically) interrupts their functions^9^. These intracellular modifications can also indirectly (epigenetically) alter cell behaviours^6,10^ and, worse, cause apoptosis^11^. Therefore, DMSO is indispensable but not a be-all end-all solvent in the life sciences.

**Fig. 1:**
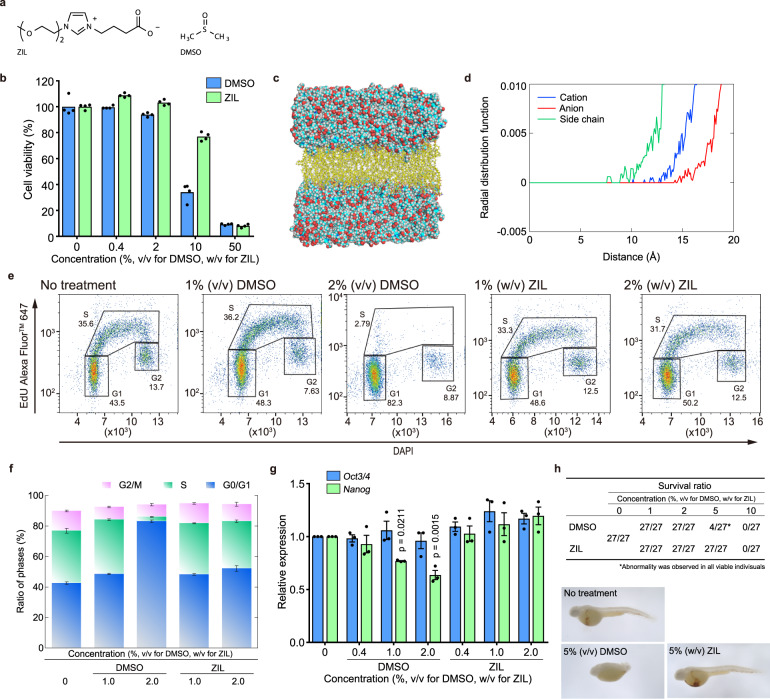
ZIL possesses higher biocompatibility than DMSO. **a** Structures of solvents. **b** Cell viability of hNF-1 cells cultured with DMSO or ZIL for 24 h at the indicated concentrations (*n* = 4, experimental quadruplicate). **c** An overlaid image showing that ZIL molecules (spheres) do not pass through the cell membrane produced by MD simulation for 2.5 µs. Lipid molecules (sticks) of the final structure are displayed, and water molecules are omitted for clarity. **d** Radial distribution functions of the indicated part of ZIL from the centre of gravity of the cell membrane (i.e. midpoints of double layer) in 5 wt% ZIL solution. **e**, **f** Cell cycle analyses and their quantitative results (*n* = 3, independent experiments) with 5-ethynyl-2′-deoxyuridine (EdU) incorporation and 4′,6-diamidino-2-phenylindole (DAPI) staining in MDA-MB-231 cells treated with DMSO or ZIL for 24 h at the indicated concentrations. **g** Expression of *Oct3/4* and *Nanog* in human iPS cells after treatment with DMSO or ZIL at the indicated concentrations for 24 h (*n* = 3, independent experiments). **h** Survival ratio of zebrafish embryos at 24 hpf after treatment with DMSO or ZIL and appearance of zebrafish embryos at 48 hpf after treatment with 5% (v/v) DMSO or 5% (w/v) ZIL at the indicated concentrations. The embryos were stained with *o*-dianisidine to check erythropoiesis. All error bars indicate standard error.

Recently, we reported a solvent that has a zwitterionic structure (zwitterionic liquid—zwitterion-type ionic liquid; ZIL), whose modules are similar to histidine shown in Fig. 1a^12^. ZIL exists as a liquid at ambient temperature, while almost all zwitterions, including amino acids, are solids. Notably, ZIL dissolves cellulose and related materials that exhibit very low solubility in water and DMSO, indicating strong potential as a nonaqueous solvent. In addition, ZIL shows little toxicity to *Escherichia coli* (*E. coli*), which enables “one-pot” ethanol synthesis from plant biomass^12,13^. However, ZIL does not have any positive effect for the culture of *E. coli* in the previous cases—ZIL merely does not adversely affect behaviours of *E. coli* at the microscopic scale such as growth. We here discover the further biocompatibility of ZIL based on the molecular mechanisms and suggest ZIL as a functionalised solution superior to DMSO, serving as a cryoprotectant and a vehicle for hydrophobic drugs, for instance. Animal cells and tissues are used in this research to suggest practical feasible applications. In this study, the ZIL exhibited lower toxicity than DMSO from viewpoints of cell viability and cell behaviours. The ZIL dissolved hydrophobic drugs including DMSO-insoluble drugs. Furthermore, ZIL enabled preparation of stock solutions of platinating agents, whose anticancer effects are completely abolished by dissolution in DMSO. The ZIL-containing freezing media cryopreserved animal cells with high efficiency. These results indicate the ZIL will take over a part of roles of DMSO in the life science.

## Results and discussion

### Effect of ZIL on cells and tissues

We found that ZIL shows less toxicity to human cells (normal fibroblasts, hNF-1) than DMSO at high (10%) concentrations (Fig. 1b). It is here noted that the ZIL solutions were prepared based on weight per volume, although the DMSO solutions were based on volume per volume, the most general method in the life sciences. The reason is that we wanted to prepare solutions with precise concentrations—ZIL is too viscous to measure its precise volume at small scales. We found that structurally similar ions scissored at one or two points (Supplementary Fig. 1a) exhibited higher toxicity than ZIL (Supplementary Fig. 1b), suggesting that the zwitterionic structure is critical for cell compatibility. In addition, mass spectrometry analysis with electrospray ionisation revealed that the intracellular concentration of ZIL was 10^−4^–10^−3^% (w/v) after 2 h of cultivation in 5% (w/v) ZIL solution, while the intracellular DMSO concentration was reported to be approximately 1.6% (w/v) after 2 h of cultivation in 7.8% (w/v) DMSO solution^14^, indicating that ZIL is a non-cell-permeable reagent. Consistent with these results, molecular dynamics (MD) simulations clearly showed that no ZIL molecules passed through the cell membrane (Fig. 1c, d and Movie 1). We also confirmed that ZIL is less toxic than DMSO to another human normal fibroblast line (hNF-2) and a mouse normal fibroblast line (mNF) (Supplementary Fig. 1c).

Next, we examined the effects of DMSO and ZIL at low concentrations in cell culture experiments. We found that the addition of DMSO at a final concentration of 1–2% (v/v) suppressed cell cycle progression in all tested cell lines, while ZIL had little or no effect (Fig. 1e, f and Supplementary Fig. 2). It has been reported that 1–2% (v/v) DMSO increases the proportion of early G1-stage cells via the dephosphorylation of retinoblastoma protein (Rb) in various types of cells, including induced pluripotent stem (iPS) cells^9,15^; thus, we examined the phosphorylation status of Rb in these cells. As expected, we found that 1–2% (v/v) DMSO dephosphorylated Rb, while ZIL had little or no effect (Supplementary Fig. 3). Surprisingly, although ZIL and DMSO exhibited similar toxicity to human iPS cells and their feeder cells at high concentrations (Supplementary Fig. 4), low-dose ZIL did not affect the expression levels of undifferentiated markers (*Oct3/4* and *Nanog*), while DMSO downregulated them in a dose-dependent manner (Fig. 1g) as reported previously^15^. Because most polar solvents, such as *N,N*-dimethylacetamide and triethylene glycol, are also reported to cause differentiation of some cells^16^, our results suggest that ZIL possesses high potential for application in stem cell research. Finally, we tested the impact of DMSO and ZIL in living tissues using zebrafish development as an experimental model. DMSO is known to cause malformation in rodent embryos^17^, and we found that most of the zebrafish embryos treated with 5% (v/v) DMSO from 1.5 h post-fertilisation (hpf) died before 24 hpf, while 4 out of 27 embryos survived with severe malformation (Fig. 1h and Supplementary Fig. 5). In contrast, all embryos treated with 5% (w/v) ZIL from 1.5 to 24 hpf survived without malformation. Treatment with 5% (w/v) ZIL also did not affect haemoglobin production, as evidenced by *o*-dianisidine staining, confirming the lack of effect of ZIL treatment on erythropoiesis (Fig. 1h and Supplementary Fig. 5). These results indicate that ZIL is less toxic even in embryogenesis. Taken together, these results strongly suggest that ZIL possesses higher biocompatibility than DMSO and has great potential for application in the life sciences.

### ZIL as a vehicle for hydrophobic compounds

**Fig. 2:**
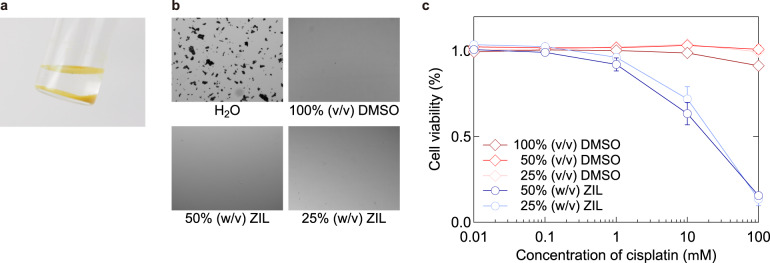
ZIL aq. serves as a vehicle for cisplatin stock solution. **a** Megascopic view of cisplatin (1 wt%) in water. **b** Microscopic observation of 10 mM cisplatin stock solution dissolved in the indicated solvents. **c** Cell viability assay of MDA-MB-231 cells treated with cisplatin for 72 h at the indicated concentrations (*n* = 3, independent experiments). Cisplatin stock solutions were prepared with the indicated solvents at least 24 h prior to the experiments. All error bars indicate standard error.

The high biocompatibility of ZIL motivated us to examine whether it can also serve as a vehicle for various compounds, as DMSO serves as a good solvent for hydrophobic drugs. We tested 12 hydrophobic compounds (the structures are shown in Supplementary Fig. 6a) and found that 8 of them were solubilised in ZIL and/or ZIL aqueous solution (ZIL aq.) (50, 25, or 5%, w/v), including zoledronic acid monohydrate, which is soluble in neither water nor DMSO (Supplementary Fig. 6b). Interestingly, ZIL aq. solubilised adenosine 3′-phosphate and insulin, which are soluble in neither 100% ZIL nor DMSO (Supplementary Fig. 6b). DMSO is a polar organic solvent and dissolves a wide range of drugs from relatively polar drugs to relatively non-polar drugs. On the other hand, water dissolves only polar drugs such as adenosine 3′-phosphate. ZIL is also a polar solvent and seems to dissolve polar drugs. The difference in dissolution abilities of water and ZIL may be based on hydrogen bond acidity and basicity: water and ZIL have high hydrogen bond acidity and basicity, respectively^12,13^. Our results strongly suggest that although not universal, ZIL and ZIL aq. can serve as solvents for various compounds that are not soluble in water and/or DMSO. Here, it should be noted that ZIL and ZIL aq. dissolve platinating agents that are not soluble in water (Fig. 2a, b). Importantly, the anticancer effects of platinating agents (especially cisplatin) are almost completely abolished by solvolysis in DMSO^5,18^. Therefore, these drugs cannot be stocked at high concentrations and must be prepared as low-scale working solutions when needed. We tested 50 and 25% (w/v) ZIL aq. and found that they can serve as a solvent for a 10 mM stock solution of cisplatin without affecting its anticancer activity (Fig. 2c). We found that cisplatin dissolved in DMSO aqueous solution (DMSO aq.) (50 and 25%, v/v) also lost its cytotoxicity, indicating that the number of DMSO molecules is far excess against cisplatin molecules even after dilution, which causes solvolysis of the drug. Here, we suggest ZIL aq. as the first workable solvent for stock solutions of platinating agents because any solvents other than DMSO also do not properly work^5^.

### ZIL as a cryoprotectant of cells

**Fig. 3:**
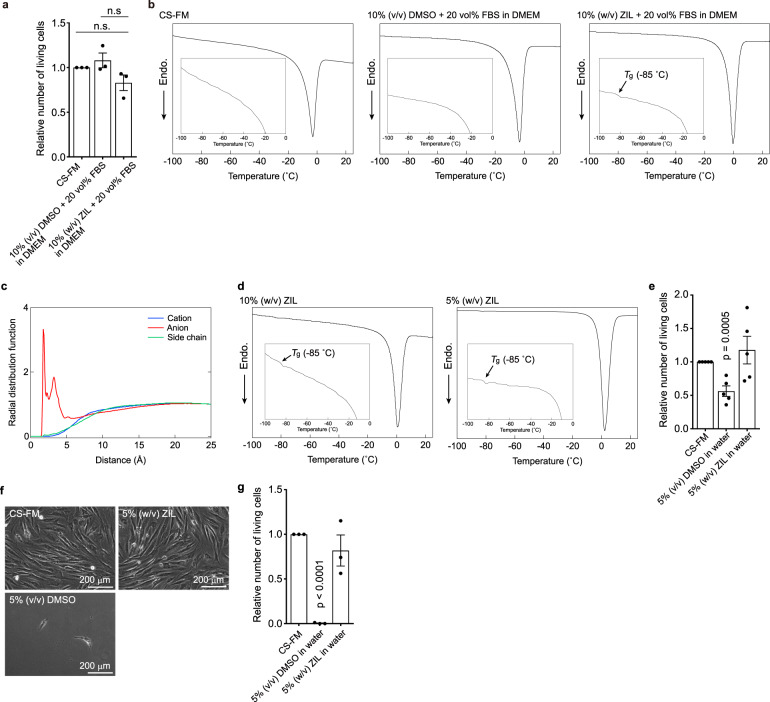
ZIL serves as a cryoprotectant because of its glass forming ability and compatibility to cell membrane. **a** Relative number of living hNF-2 cells stocked at –85 °C for 3 days in the indicated freezing media (*n* = 3, biologically independent samples). CS-FM: CultureSure freezing medium (Fujifilm Wako pure chemical corporation), which contains DMSO and BSA. **b** DSC charts of the indicated solutions (the arrows indicate the glass transition temperature). **c** Radial distribution functions of the indicated part involving ZIL from the amine head group of the lipid component contained in the cell membrane in 5 wt% ZIL solution at 37 °C. **d** DSC charts of the indicated solutions (the arrows indicate the glass transition temperature). **e** Relative number of living hNF-2 cells stocked at –85 °C for 3 days in the indicated freezing media (*n* = 5, biologically independent samples). **f**, **g** Microscopic evaluation and relative number of living hNF-2 cells 24 h after recovery from freezing (*n* = 3, biologically independent samples). All error bars indicate standard error.

DMSO is also used as a universal cryoprotectant, which strongly interacts with water molecules and prevents ice crystal formation^19^. From the chemical structure, we expected ZIL to be another good cryoprotectant since ZIL is an aprotic polar solvent similar to DMSO. Furthermore, ZIL should interact with water molecules more strongly than DMSO because ZIL has higher hydrogen bond basicity than DMSO^13^. Therefore, we first tested whether ZIL can directly replace DMSO in freezing medium. Typically, freezing medium in laboratory research consists of 10 vol% DMSO in Dulbecco’s modified Eagle’s medium (DMEM) containing 20 vol% foetal bovine serum (FBS). We compared the viabilities of hNF-2 freeze-stocked for 3 days at –85 °C in DMEM/FBS/DMSO (70/20/10, v/v/v) and DMEM/FBS/ZIL (70/20/10, v/v/w). DMEM/FBS/ZIL clearly exhibited cryoprotective effects like a commercially available freezing medium, CultureSure freezing medium® (CS-FM, containing DMSO and bovine serum albumin (BSA)) and DMEM/FBS/DMSO (Fig. 3a). On the other hand, because DMSO is a cell-permeable cryoprotectant and considered to exert its cryoprotective effect both intra- and extracellularly, non-cell-permeable ZIL theoretically cannot completely replace DMSO. Therefore, ZIL may adequately dehydrate the cells to prevent intracellular ice formation like other non-cell-permeable cryoprotectants^20–22^. It is also reported that non-cell-permeable cryoprotectants can exert their efficacy by stabilising the plasma membranes^23,24^; however, it is totally unclear at this stage whether ZIL has similar effect as these non-cell-permeable cryoprotectants. Differential scanning calorimetry (DSC) showed a large melting signal at approximately 0 °C in DMEM/FBS/ZIL, clearly suggesting that a major part of the solution turned to ice, as is usually observed in slow-freezing conditions. In addition, a tiny glass transition signal was also observed in the DSC chart, indicating that the solution contains unfrozen compartment (Fig. 3b). These results suggest that ZIL might gradually be concentrated near the surface of cells while ice crystals grow below 0 °C, and then the concentrated ZIL solution may adequately dehydrate the cells. It is unclear at this stage whether the glass transition of the unfrozen part is involved with the cryoprotecting effect. MD simulation supports that the ZIL was concentrated near the surface of cells—ZIL molecules, especially their carboxylates, electrostatically interact with the cell membrane, and consequently, ZIL is concentrated ~3 times more densely on the polar surface of the cell membrane than in the bulk state, even at 37 °C (Fig. 3c and Supplementary Fig. 7). In addition, Matsumura and co-workers^25^ have reported that polyampholytes which possess strong affinity to plasma membranes act as good cryoprotectans. ZIL might cryopreserve cells through a similar mechanism to the polyampholyte-type cryoprotectants but further investigation should be needed.

The unique cryoprotectant further inspired us to apply ZIL aq. itself as an extremely simple freezing medium. We confirmed that 10 and 5% (w/v) ZIL aq. showed similar DSC profiles to that of DMEM/FBS/ZIL (Fig. 3d): a major part (92 wt%) of the solution turned to ice crystals and the minor part (8 wt%; 5 wt% ZIL + 3 wt% water) of the solution was not frozen under subzero temperature (Supplementary Figs. 8 and 9). Then, we tested 5% (w/v) ZIL aq., which does not show significant cell toxicity (see Fig. 1b and Supplementary Fig. 1), and surprisingly found that we could cryopreserve hNF-2, while freezing in 5% (v/v) DMSO aq. severely damaged the cells (Fig. 3e–g). Finally, we examined whether 5% (w/v) ZIL aq. can generally serve as a freezing medium for other cell types. Although the cryopreservation efficacy differs between cell types, 5% (w/v) ZIL aq. served as a freezing medium for various cell types (Supplementary Fig. 10). Five percent (w/v) ZIL aq. successfully cryopreserved hNF-2 for 1 year with a similar efficiency given by CS-FM (Supplementary Fig. 11). Given that the cells were stocked in an extremely simple aqueous solution, there is apparently room for improvement in ZIL aq.-based freezing media. As mentioned above, general freezing media contain DMSO, FBS or BSA and other additives to optimise pH and osmotic pressure. As is often noted, there is still a large unmet need for an alternative cryomedium to replace all of these components, especially DMSO, FBS and BSA^26^. In this context, ZIL aq.-based freezing media have great potential to fulfil this need. Furthermore, ZIL aq.-based freezing media will provide some additional advantages: (1) the composition can be completely chemically defined, (2) the synthetic route is a completely chemical procedure, thus reducing the risk of unexpected infection, (3) the cost is low (~2 USD/100 mL 5% ZIL aq. vs ~100 USD/100 mL for commercial cryoprotectants) and (4) they are polypeptide- and protein-free media. We expect that these advantages should especially contribute to and accelerate tissue engineering and regenerative medicine, where only defined chemicals are preferred for large-scale use.

Here, we propose ZIL as a potent, multifunctional and biocompatible solvent for use in the life sciences, which we also confirmed to be applicable to general plastic tools in biological and biochemical laboratories (Supplementary Fig. 12). ZIL may not displace DMSO from the life sciences in the near term because decades of literature and precedence will favour continued use of DMSO at dilutions that are not problematic. Thus, ZIL will be first used in applications where DMSO has adverse effects. Notably, ZIL aq. will be the first-in-class solvent for stock solutions of platinating agents. In addition, a better alternative to DMSO is needed in metabolomics^27^, epigenetics^6,10^ and embryology^17,28^. ZIL will also reveal the synergistic effects of DMSO in medicine, whose confirmation is quite difficult because there are no other biocompatible solvents equivalent to DMSO. Moreover, the development of ZIL aq.-based freezing media will have a great impact on future clinical applications. It is also noted that ZIL has some additional potential characteristics for clinical applications: ZIL is suit for production at Xeno-free and GMP-grade because ZIL is a completely synthetic chemical and easy to synthesis. In a wide range of basic science research, alternatives or equivalents to DMSO have long been desired. We expect ZIL to take this role and reduce the dependence of the life sciences on DMSO.

## Methods

### Cells

Human normal fibroblast-1 (hNF-1, KF-4009) was purchased from Kurabo Industries Ltd. Human normal fibroblast-2 (hNF-2) and mNF were kind gifts from Prof. Erik Sahai (The Francis-Crick Institute, UK) and described previously^29^. MDA-MB-231 human breast cancer cells and WM266.4 human melanoma cells are also kind gifts from Prof. Erik Sahai. PC9 human lung cancer cells and Mardin-Darby canine kidney cells are kind gifts from Prof. Seiji Yano (Cancer Research Institute of Kanazawa University) and Prof. Etsuko Kiyokawa (Kanazawa Medical University), respectively. Mouse primary astrocytes were established from euthanized neonatal C57BL6 mice (P1-3, male and female) as reported previously^30^. All cells except for hNF-1 were grown and maintained as monolayer cultures at 37 °C in 5% CO_2_ in a humidified atmosphere, using DMEM (high glucose with l-glutamine and phenol red, Fujifilm Wako Pure chemical corporation), supplemented with penicillin–streptomycin solution (×100) (Fujifilm Wako Pure Chemical Corporation), and 10 vol% FBS (Sigma-Aldrich Co., Llc.). The cells were sub-cultured every 4–6 days using trypsin solution (0.5 w/v% trypsin—5.3 mmol/L EDTA·4Na solution without phenol red (×10), Fujifilm Wako Pure Chemical Corporation). hNF-1 was grown and maintained as monolayer cultures at 37 °C in 5% CO_2_ in a humidified atmosphere, using DMEM (low glucose with l-glutamine and phenol red, Sigma-Aldrich Co., Llc.) and 5 vol% FBS (Thermo Fisher Scientific Inc.). hNF-1 were sub-cultured every month using trypsin solution (0.25%, Nacalai Tesque, Inc.) prepared with Dulbecco’s phosphate-buffered saline (DPBS, no calcium, no magnesium, Thermo Fisher Scientific Inc.).

### Investigation of ZIL content in the cells

ZIL was synthesised as reported^12^. Around 5 × 10^5^ cells were incubated in 5% (w/v) ZIL in DMEM after 2 h. The cells were mechanically crushed by using a cell scraper in 2 mL of methanol after six times PBS washing (2 mL each). The solutions were centrifuged and filtrated before subjecting to mass spectrometry (JMS-T100TD, JEOL Ltd) through electrospray ionisation. The volume of the cells was calculated by measuring the radii of the cells under microscopic observation by an IX83 inverted microscope (Olympus Corporation).

### Toxicity of solvents to cells

ZIL analogue 1 was synthesised as reported^12^. ZIL analogue 2 was purchased from Iolitech GmbH and used after drying. hNF-2 and mNF were seeded in 96-well plates with 4 × 10^3^ cells in each well. The solvents were added to each medium at indicated concentrations. After cultivation for 24 h, cell viability was investigated with CellTiter 96® Aqueous One Solution (Promega Corporation). hNF-1 was seeded in 96-well plates with 2 × 10^4^ cells in each well. After 24 h cultivation in the media/solvent mixtures at the indicated concentrations, the culture medium was changed to another medium supplemented with 33 mg/mL neutral red. After 2 h cultivation, the cells were washed with PBS and the neutral red was extracted with 0.1 M HCl–30% ethanol solution. The cell viability was calculated from the absorbance at 650 nm (derived from neutral red) and 550 nm (derived from cells themselves).

### MD simulation

The cell membrane in the simulation system was composed of dioleoylphosphatidylethanolamine (DOPE)–dioleoylphosphatidylglycerol (DOPG) lipids with a 3:1 ratio (90 and 30 molecules, respectively). Lipid bilayer was placed at centre in a rectangular periodic box filled with TIP3P water and ZIL (with 8716 and 32 molecules, respectively), where the buffer size between the edges of the box and the lipid model was set at around 34 Å, and the MD simulation of cell membrane in 5 wt% ZIL aqueous solution was carried out using Amber18 and AmberTools19 software^31^.

The cell membrane system was optimised and heated gradually from −253 to 37 °C at 0.1 °C/ps. This was followed by production dynamics at a constant temperature (37 °C) and pressure (1 bar) for 2.5 µs. Lipid molecules were described by the Lipid17 force field^32^ and ZIL molecules were modelled using the GAFF parameters. The MD simulation was performed using a 2 fs integration time step coupled with the SHAKE option. The particle mesh Ewald (PME) method was adopted for long-range interactions, and the cutoff for nonbonding interactions in the coordinate space was fixed at 10 Å. The atoms used for the calculations are shown in Supplementary Fig. 7.

### Cell cycle analysis

5-Ethynyl-2′-deoxyuridine (EdU) incorporation assay was performed by using Click-iT Plus EdU Flow Cytometry Assay Kits (Thermo Fisher Scientific Inc.) following the manufacturer’s protocol. Briefly, cells were incubated with 10 μM EdU for 1 h. Then, the cells were trypsinised, fixed with 4% PFA, permeabilised and labelled with Alexa Fluor 647 picolyl azide. Then, the cells were stained with 4′,6-diamidino-2-phenylindole (DAPI) and analysed with BD FACSAria^TM^ III (BD Biosciences).

### Immunoblotting of phospho-Rb

Protein lysates were processed following standard procedures and analysed by SDS-PAGE followed by immunoblotting. Precast SDS-polyacrylamide gels (4–15% Mini-PROTEAN TGX Precast Gel) and Trans-Blot Turbo transfer system were purchased from Bio-Rad. The bound antibodies were detected with secondary antibodies conjugated with IRDye680 or IRDye800 and analysed with an Odyssey Imager system (LI-COR. Inc.). The following antibodies were used in this study: rabbit anti-phospho-Rb (Ser780) antibody (9307) from Cell Signaling Technology and mouse anti-β-tublin antibody (T7816) from Sigma-Aldrich Co., Llc.

### Cell culture: iPS cell

Human iPS cell line 201B7 was provided by the RIKEN BRC through the National BioResource Project of the MEXT/AMED, Japan. The cell line was grown and maintained by culturing with feeder cells (SL10, Reprocell Inc.) at 37 °C in 5% CO_2_ in a humidified atmosphere, using Primate ES cell medium (Reprocell Inc.), supplemented with bFGF (Fujifilm Wako Pure Chemical Corporation).

The cells were sub-cultured, when the cells are 80% confluent, using a cell scraper (AGC Techno Glass Co., Ltd) after processing by DPBS(−) (no calcium, no magnesium, Thermo Fisher Scientific Inc.) and CTK solution. CTK solution (0.25% trypsin + 1 mg/mL collagenase IV + 1 mM CaCl_2_ + 20% KSR in DPBS (−)) was prepared with trypsin ((2.5%), no phenol red, Thermo Fisher Scientific Inc.), collagenase (type IV, powder, Thermo Fisher Scientific Inc.), calcium chloride dihydrate (Fujifilm Wako Pure Chemical Corporation), Knock Out^TM^ Serum Replacement (KSR, Thermo Fisher Scientific Inc.).

### Toxicity of ZIL and DMSO to iPS cells

iPS cells were removed from dishes at 80% confluence using CTK solution and washed DPBS (−). The removed cells were treated with 0.5× TrypLE Select solution. 0.5× TrypLE Select solution was prepared with 50% TrypLE Select, no phenol red (Thermo Fisher Scientific) and 0.25 mM EDTA (0.5 mol/L EDTA Solution (pH 8.0), Nacalai Tesque Inc.) in DPBS (−) for single-cell culture. The cells were collected in the medium with 10 μM Y-27632 (Fujifilm Wako Pure Chemical Corporation) and centrifuged. The supernatant was removed before use.

Feeder cells were seeded with 1.25 × 10^4^ cells/well in a 96-well plate and cultured for 1 day in the medium including bFGF and Y-27632, and iPS cells were additionally seeded with 5000 cells/well and cultured for 1 day. The culture media was substituted by those supplemented with DMSO (CultureSure DMSO, Fujifilm Wako Pure Chemical Corporation) or ZIL. The cells were cultured until the control sample (which does not contain the solvents) became 80% confluent. The cell viability was confirmed by using Cell Count Reagent SF (Nacalai Tesque Inc.). The culture media was substituted by the media supplemented with 10% of Cell Count Reagent SF and incubated for 1 h. After incubation, absorption at 450 nm was measured and translated to the cell viability with a standard curve.

### Quantification of undifferentiated markers

Feeder cells were seeded with 1.25 × 10^4^ cells/well in a 96-well plate and cultured for 1 day, and iPS cells were additionally seeded with 5000 cells/well and cultured for 1 day. The culture media was substituted by those including DMSO or ZIL and the cells were cultured until the control sample (which does not contain the solvents) became 80% confluent. The total RNA was extracted and converted to cDNA by using FastLane Cell cDNA kit (Qiagen). The expression of *Nanog* and *Oct3/4* was quantified by using LightCycler® 96 Real-Time PCR System (Roche) and SYBR Premix Ex Taq II (Tli RNaseH Plus) (95 °C, 5 s; 60 °C, 30 s; 40 cycles). *Gapdh* was used as an internal control. Primer sequences are as follows: *Nanog* forward: GACAGGGGGAGGGGAGGAGCTAGG, reverse: CTTCCCTCCAACCAGTTGCCCCAAAC; *Oct3/4* forward: CAACTGGCCGAAGAATAGCA, reverse: TCCCTGGTGGTAGGAAGAGTAAA; *Gapdh* forward: CATCCCTGCCTCTACTGGCGCTGCC, reverse: CCAGGATGCCCTTGAGGGGGCCCTC.

### Toxicity of solvents to zebrafish embryos

All animal experiments were performed in accordance with a protocol approved by the committee on animal experimentation of Kanazawa university. The zebrafish wild-type strain, AB*, was raised in a circulating aquarium system (AQUA) at 28.5 °C in a 14/10 h light/dark cycle and maintained in accordance with guidelines of the committee on animal experimentation of Kanazawa university. Embryos were incubated in E3 medium (5 mM NaCl, 0.17 mM KCl, 0.33 mM CaCl_2_, 0.33 mM MgSO_4_) containing DMSO or ZIL from 1.5 to 24 hpf. Embryos were then washed twice with E3 medium and were treated with 1-phenyl-2-thiourea (Fujifilm Wako Pure Chemical Corporation) to prevent pigmentation. Embryos at 48 hpf were stained with 0.6 mg/mL of *o*-dianisidine (Tokyo Chemical Industry Co., Ltd) for 15 min, followed by fixation with 4% paraformaldehyde (Fujifilm Wako Pure Chemical Corporation). Embryos were then washed twice with 0.1% Tween-20 (Sigma-Aldrich Co., Llc.) in PBS. Visible light imaging of embryos was captured using an Axiozoom V16 microscope (Carl Zeiss AG) with a TrueChrome II digital camera (BioTools Inc.) and TCapture software (ver. 4.3.0.602) (Fuzhou Tucsen Photonics Co., Ltd).

### Solubility assay of drugs

The drugs (1 wt%) were added to the solvents and stirred over 10 h at room temperature. When the drugs were not solubilised, the solutions were heated up to 80 °C and stirred for 2 h. We checked whether the drugs were solubilised by observing the particles of the drugs using an invert microscope (ECLIPSE Ts2, Nikon Corporation). Erythromycin, 3′-azido-3′-deoxythymidine, 5-iodo-2′-deoxyuridine, (+)-catechin hydrate, l-adrenaline, desloratadine, zoledronic acid monohydrate, testosterone, estriol, and oxaliplatin were purchased from Tokyo Chemical Industry Co., Ltd and used as received. Fluorouracil, carboplatin and cisplatin were purchased from Fujifilm Wako Pure Chemical Corporation and used as received. Adenosine 3′-phosphate and insulin were purchased from Nacalai Tesque Inc. and used as received. l-Thyroxine was purchased from Sigma-Aldrich Co., Llc. and used as received. Paclitaxel was purchased from Funakoshi Co., Ltd and used as received.

### Cell viability assay with cisplatin

MDA-MB-231 were seeded with 5000 cells/well in a 96-well plate and pre-cultured for 24 h. Cisplatin solution (10 mM) was prepared by using 25 or 50% (w/v) ZIL or 100, 50 or 25% (v/v) DMSO solutions and left for more than one day. They were added to the media to be the intended concentrations. After 72 h cultivation, the cell viability was investigated with CellTiter 96® Aqueous One Solution.

### Cryopreservation

The DMSO and ZIL solutions were prepared by mixing with ultrapure water or DMEM/FBS. Cells (1 × 10^6^ cells) were collected in 1.5 mL sampling tubes (Watoson Co., Ltd) and centrifuged. After removing the supernatant, 100 μL of freezing media were added and pipetted slowly. The samples were stored for 3–5 days in a box (Mr. Frosty, Thermo Fisher Scientific Inc.) for cooling at around –1 °C/min in a –85 °C freezer. The frozen samples were thawed by adding 37 °C culturing media. The number of living cells were counted by using an automated cell counter (Countess II, Thermo Fisher Scientific Inc.) after staining with trypan blue by pipetting for around 10 s. Relative number of living cells is defined as the following equation:

relative number of living cells = counted living cell number (sample)/counted living cell number (control),

where the control is cells cryopreserved by a commercially available cryoprotectant, CultureSure freezing medium (CS-FM, Fujifilm Wako Pure Chemical Corporation). Then, the cells were seeded onto six-well plate, cultured for 24 h and imaged with an inverted microscope (IX83, Olympus). After the imaging, the cells were trypsinised, re-suspended in the complete media, and relative number of living cells were counted by using Countess II after staining with trypan blue. The phase behaviour of the solutions was investigated by DSC (DSC-60A plus, Shimadzu Corporation).

### Statistical analysis

Data were subjected to one-way ANOVA analysis, followed by Dunnett’s multiple comparison test. When two groups were compared, a two-tailed unpaired Student’s *t*-test was applied.

### Reporting summary

Further information on research design is available in the Nature Research Reporting Summary linked to this article.

## Supplementary information

Reporting Summary

Supplementary Information

Description of Additional Supplementary Files

Supplementary Movie 1

## Data Availability

Data that support the findings of this study are available upon reasonable request from the corresponding authors.
