# Supplementary material for: Non-aqueous, zwitterionic solvent as an alternative for dimethyl sulfoxide in the life sciences
Source: Commun Chem. 2020 Nov 11;3:163. doi: 10.1038/s42004-020-00409-7 (PMC9814479; doi:10.1038/s42004-020-00409-7)
Supplement: Supplementary file 1 — Reporting Summary [file 42004_2020_409_MOESM1_ESM.pdf]

nature research

Corresponding author(s): Kosuke Kuroda, Eishu Hirata

Last updated by author(s): Sep 26, 2020

Reporting Summary

Nature Research wishes to improve the reproducibility of the work that we publish. This form provides structure for consistency and transparency in reporting. For further information on Nature Research policies, see our Editorial Policies and the Editorial Policy Checklist.

Statistics

For all statistical analyses, confirm that the following items are present in the figure legend, table legend, main text, or Methods section.

n/a	Confirmed	
☐	☒	The exact sample size (n) for each experimental group/condition, given as a discrete number and unit of measurement
☐	☒	A statement on whether measurements were taken from distinct samples or whether the same sample was measured repeatedly
☐	☒	The statistical test(s) used AND whether they are one- or two-sided<br>*Only common tests should be described solely by name; describe more complex techniques in the Methods section.*
☒	☐	A description of all covariates tested
☒	☐	A description of any assumptions or corrections, such as tests of normality and adjustment for multiple comparisons
☐	☒	A full description of the statistical parameters including central tendency (e.g. means) or other basic estimates (e.g. regression coefficient) AND variation (e.g. standard deviation) or associated estimates of uncertainty (e.g. confidence intervals)
☐	☒	For null hypothesis testing, the test statistic (e.g. F, t, r) with confidence intervals, effect sizes, degrees of freedom and P value noted<br>*Give P values as exact values whenever suitable.*
☒	☐	For Bayesian analysis, information on the choice of priors and Markov chain Monte Carlo settings
☒	☐	For hierarchical and complex designs, identification of the appropriate level for tests and full reporting of outcomes
☒	☐	Estimates of effect sizes (e.g. Cohen's d, Pearson's r), indicating how they were calculated

Our web collection on statistics for biologists contains articles on many of the points above.

Software and code

Policy information about availability of computer code

Data collection	qPCR data were acquired with LightCycler® 96 Real-Time PCR System (Roche). Cell cycle data were acquired with BD FACSAria III using BD FACSDiva v8.0.1. Phase contrast images were acquired with Olympus IX83 using cellSens Dimension software. Cell viability assay was conducted with TECAN infinite F200PRO using i-control software. Visible light images were aquired with Axiozoom V16 microscope (Carl Zeiss AG) with a TrueChrome II digital camera (BioTools Inc.) and TCapture software (ver. 4.3.0.602) (Fuzhou Tucsen Photonics Co., Ltd.). The phase behaviour of the solutions was analysed by DSC-60A plus (Shimadzu corporation) and TA-60WS software (Shimadzu corporation). Number of cells were counted by Countess II (Thermo Fisher Scientific Inc.). Drug solubility was confirmed by ECLIPSE Ts2 (Nikon Corporation).
Data analysis	Cell cycle data were analyzed with FlowJo v10. Immuno blotting results were analyzed and quantified with with Fiji (ImageJ, version 1.8.0). All the statistical data were analyzed with Microsoft Excel and/or GraphPad Prism 7.

For manuscripts utilizing custom algorithms or software that are central to the research but not yet described in published literature, software must be made available to editors and reviewers. We strongly encourage code deposition in a community repository (e.g. GitHub). See the Nature Research guidelines for submitting code & software for further information.

Data

Policy information about availability of data

All manuscripts must include a data availability statement. This statement should provide the following information, where applicable:
- Accession codes, unique identifiers, or web links for publicly available datasets
- A list of figures that have associated raw data
- A description of any restrictions on data availability

All data are available from the authors upon reasonable request.

Field-specific reporting

Please select the one below that is the best fit for your research. If you are not sure, read the appropriate sections before making your selection.

☒ Life sciences ☐ Behavioural & social sciences ☐ Ecological, evolutionary & environmental sciences

For a reference copy of the document with all sections, see nature.com/documents/nr-reporting-summary-flat.pdf

Life sciences study design

All studies must disclose on these points even when the disclosure is negative.

Sample size	Sample size was determined based on previous experience and published literature.
Data exclusions	No data were excluded from the study
Replication	Experiments were carried out with the number of replicates indicated in the manuscript. All attempts at replication were successful.
Randomization	Randomization was not necessary for the experiments.
Blinding	No blinding.

Reporting for specific materials, systems and methods

We require information from authors about some types of materials, experimental systems and methods used in many studies. Here, indicate whether each material, system or method listed is relevant to your study. If you are not sure if a list item applies to your research, read the appropriate section before selecting a response.

Materials & experimental systems

n/a	Involved in the study	
☐	☒	Antibodies
☐	☒	Eukaryotic cell lines
☒	☐	Palaeontology and archaeology
☐	☒	Animals and other organisms
☒	☐	Human research participants
☒	☐	Clinical data
☒	☐	Dual use research of concern

Methods

n/a	Involved in the study	
☒	☐	ChIP-seq
☐	☒	Flow cytometry
☒	☐	MRI-based neuroimaging

Antibodies

Antibodies used	Rabbit anti-phospho-Rb (Ser780) antibody (9307, Cell Signaling Technology)<br>Mouse anti-beta-tublin antibody (T7816, Sigma-Aldrich Co., Llc.)
Validation	Antibodies were used and stored according to the manufacturer's inscructions.

Eukaryotic cell lines

Policy information about cell lines

Cell line source(s)	Human normal fibroblast-1 (hNF-1, KF-4009) was purchased from Kurabo Industries Ltd. Human normal fibroblast-2 (hNF-2) and mouse normal fibroblast (mNF) are kind gifts from Prof. Erik Sahai. Mouse primary astrocytes were established from euthanized neonatal C57BL6 miceMDA-MB-231 human breast cancer cells and WM266.4 human melanoma cells are kind gifts from Prof. Erik Sahai (The Francis-Crick Institute, UK). PC9 human lung cancer cells and Mardin-darby canine kidney cells are kind gifts from Prof. Seiji Yano (Cancer Research Institute of Kanazawa University) and Prof. Etsuko Kiyokawa (Kanazawa Medical University), respectively. Human iPS cell line 201B7 was bought from Riken BRC.
Authentication	MDA-MB-231, WM266.4, PC9, MDCK, iPS cell lines were STR profiled and species verified before acquisition. Human normal fibroblasts (hNF-1 and hNF-2), mouse normal fibroblasts (mNF) and mouse primary astrocytes have been species verified and tested for the expected phenotype in vitro.
Mycoplasma contamination	All cells were tested negative for mycoplasma contamination.
Commonly misidentified lines (See ICLAC register)	No commonly misidentified cell lines were used.

Animals and other organisms

Policy information about studies involving animals; ARRIVE guidelines recommended for reporting animal research

Laboratory animals	Species, zebrafish; strain, AB* (wild type strain); sex and age, n/a
Wild animals	n/a
Field-collected samples	n/a
Ethics oversight	n/a

Note that full information on the approval of the study protocol must also be provided in the manuscript.

Flow Cytometry

Plots

Confirm that:

- [x] The axis labels state the marker and fluorochrome used (e.g. CD4-FITC).
- [x] The axis scales are clearly visible. Include numbers along axes only for bottom left plot of group (a 'group' is an analysis of identical markers).
- [x] All plots are contour plots with outliers or pseudocolor plots.
- [x] A numerical value for number of cells or percentage (with statistics) is provided.

Methodology

Sample preparation	Detailed protocols for cell culture and sample preparation are provided in Methods section.
Instrument	BD FACS AriaIII
Software	BD FACSDiva v8.0.1 and FlowJo v10.
Cell population abundance	As shown in the figures.
Gating strategy	SSC-A/FSC-A gate was used to remove debris and dead cells, SSC-A/SSC-H and FSC-A/FSC-H were used to remove cell doublets. Cell cycle gates were set according to the DAPI and Alexa Fluor 647 intensity as shown in the figures.

- [x] Tick this box to confirm that a figure exemplifying the gating strategy is provided in the Supplementary Information.
