# Supplementary material for: Non-aqueous, zwitterionic solvent as an alternative for dimethyl sulfoxide in the life sciences
Source: Commun Chem. 2020 Nov 11;3:163. doi: 10.1038/s42004-020-00409-7 (PMC9814479; doi:10.1038/s42004-020-00409-7)
Supplement: Supplementary file 2 — Supplementary Information [file 42004_2020_409_MOESM2_ESM.pdf]

Supplementary information

Non-aqueous, zwitterionic solvent as an alternative for dimethyl sulfoxide in the life sciences

Kosuke Kuroda, Tetsuo Komori, Kojiro Ishibashi, Takuya Uto, Isao Kobayashi, Riki Kadokawa, Yui Kato, Kazuaki Ninomiya, Kenji Takahashi, Eishu Hirata

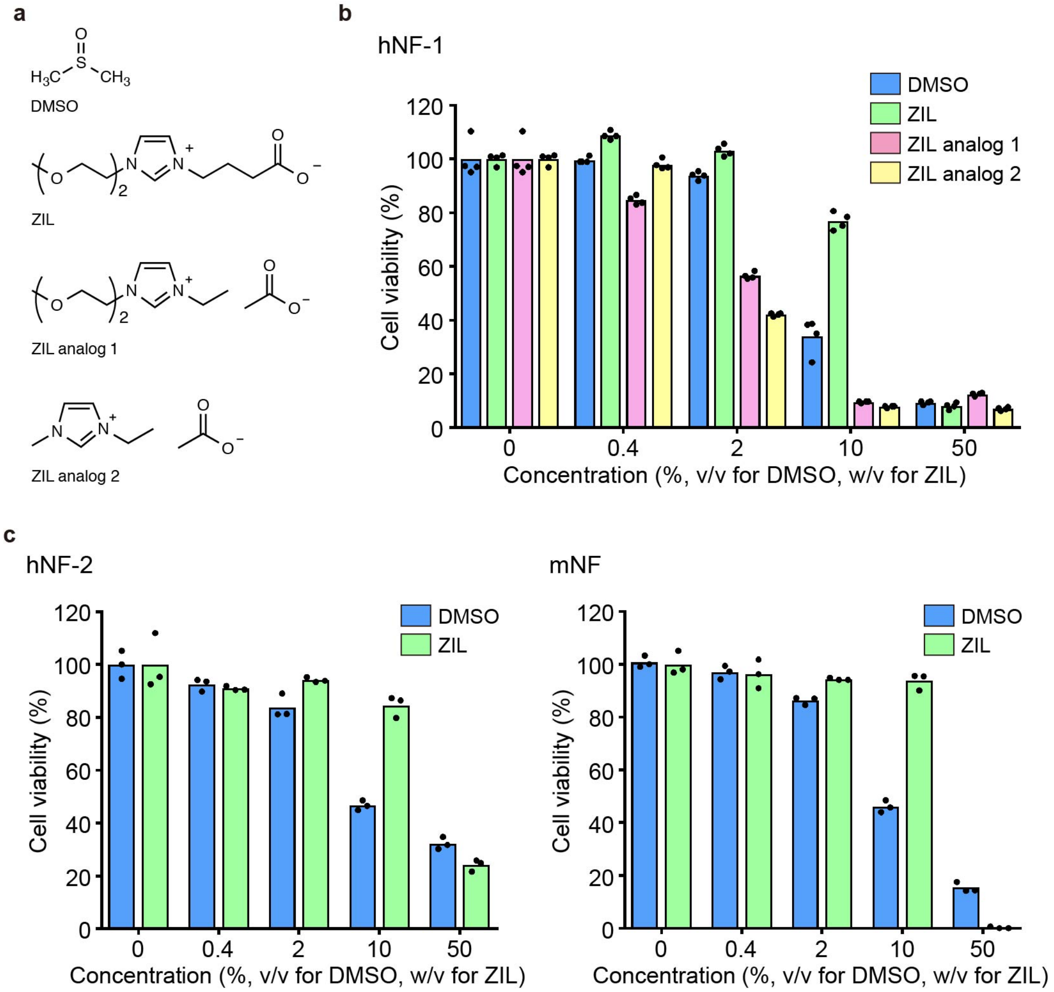

Supplementary Fig. 1 | Zwitterionic structure is critical for cell compatibility. a, Structures of solvents used in this study. **b**, Cell viability of hNF-1 cultured with the indicated solvents for 24 h at different concentrations (n = 4, experimental quadruplicate). The data regarding DMSO and ZIL are reproduced from Fig. 1b. **c**, Cell viability of human normal fibroblast-2 (hNF-2) and mouse normal fibroblast (mNF) cultured with the indicated solvents for 24 h at different concentrations (n = 3, experimental triplicate). The separated ions (ZIL analogs 1 and 2) exhibited higher toxicity than ZIL and the oligoether does not exhibit a special function. Free organic ion pairs like ZIL analogs 1 and 2 exhibit the toxicity by inserting their alkyl chains of cations into cell membrane via hydrophobic interaction[1]. ZIL does not have any hydrophobic alkyl chains due to capping by anions, and therefore its toxicity is significantly less than free ion pairs[2]. The zwitterionic structure of ZIL is confirmed to be a key factor for low toxicity.

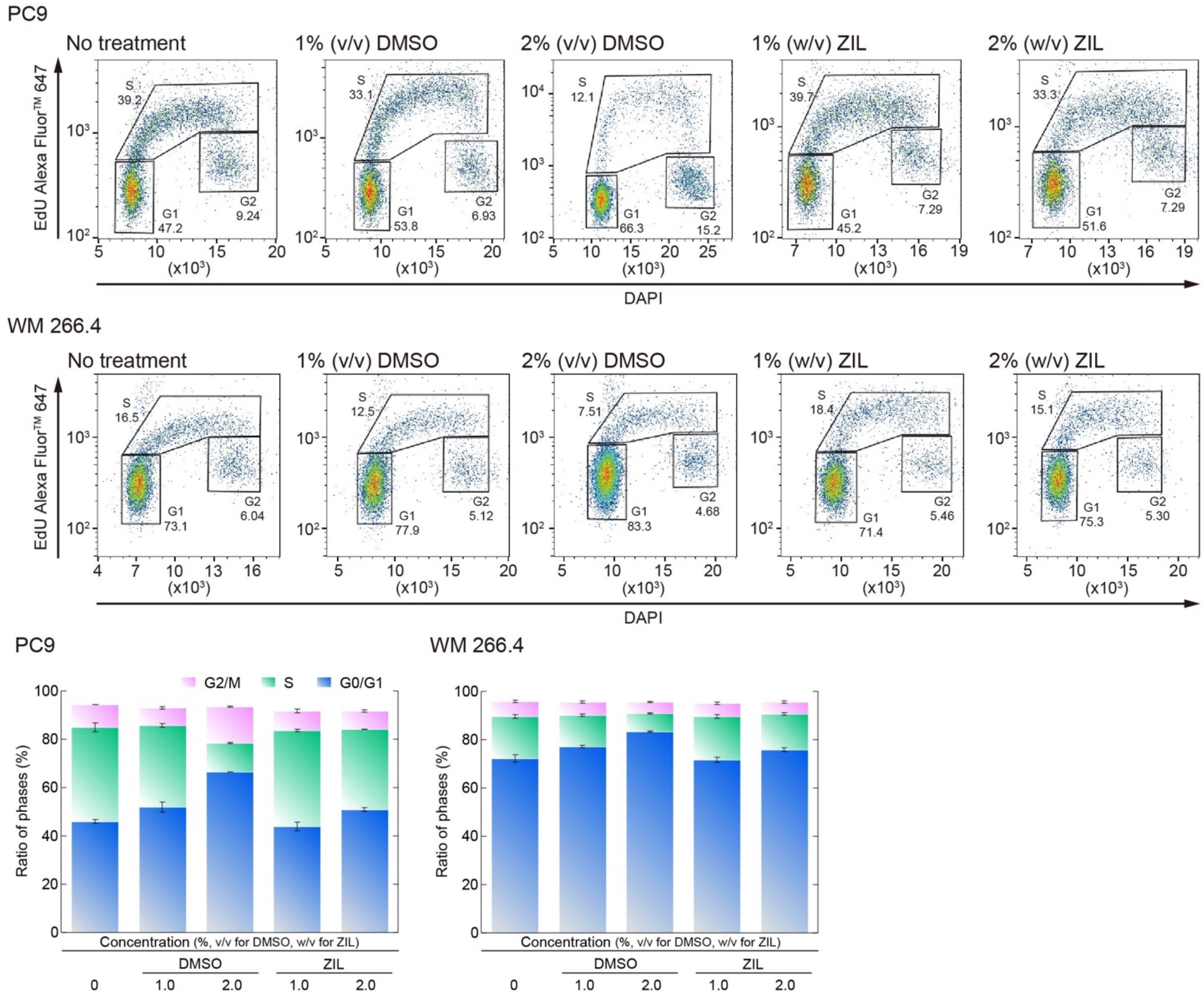

Supplementary Fig. 2 | Low doses of ZIL do not affect cell cycle progression of various cancer cells unlike DMSO. Cell cycle analysis with EdU incorporation and DAPI staining in PC9 and WM266.4 cells treated with DMSO or ZIL for 24 h at the indicated concentrations. The ratio of cells in G0/G1, S and G2/M phase are quantified and shown below (n = 3, independent experiments). All error bars indicate standard error.

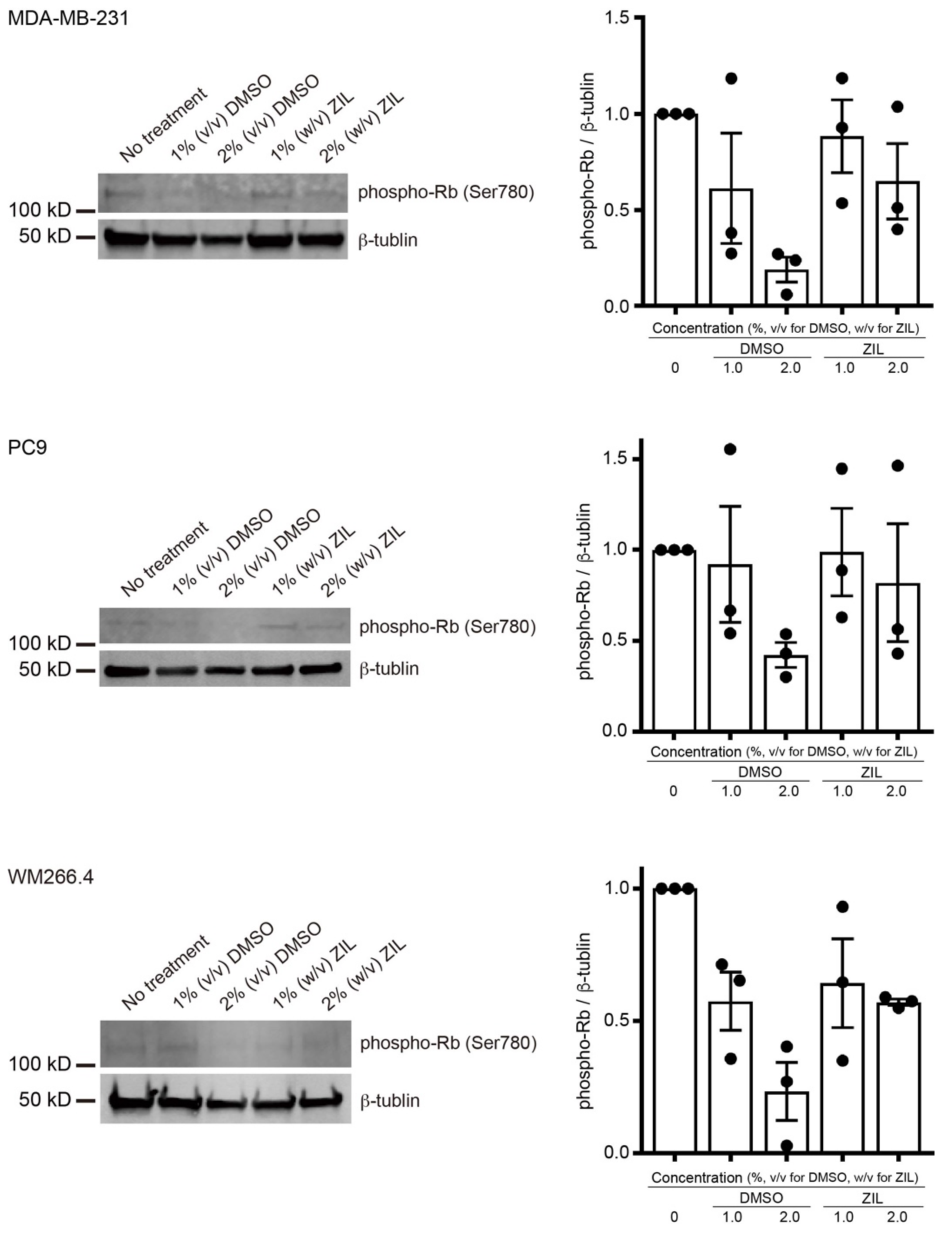

Supplementary Fig. 3 | Low doses of ZIL do not significantly dephosphorylate Rb unlike DMSO. Immunoblotting with the indicated antibodies in MDA-MB-231 cells, PC9 cells and WM266.4 cells treated with DMSO or ZIL for 24 h. The values of phospho-Rb/β-tublin are quantified and shown in the right panels (n = 3, independent experiments). All error bars indicate standard error.

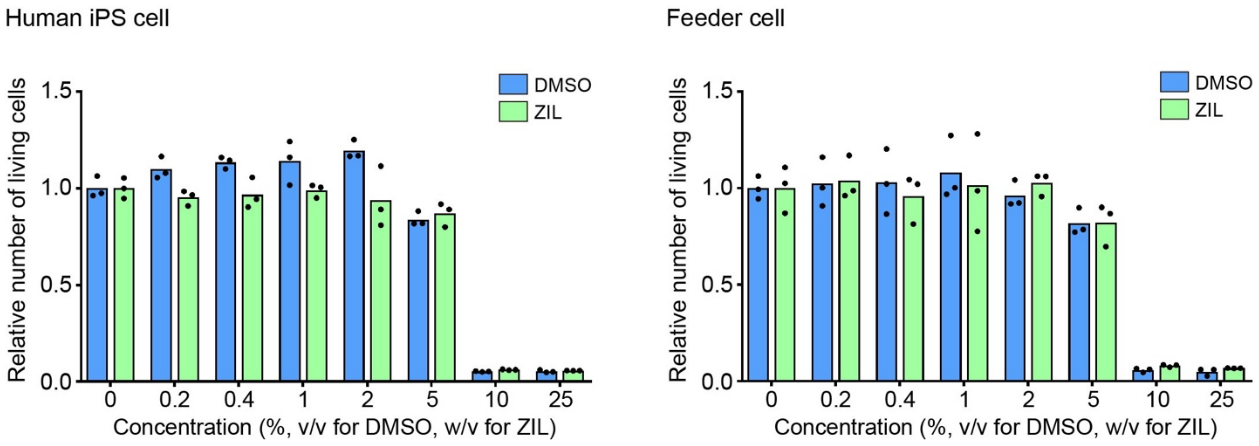

Supplementary Fig. 4 | DMSO and ZIL possess similar compatibility to human iPS cells and the feeder cells. Cell viability of human iPS cells and the feeder cells cultured with DMSO or ZIL for 24 h at the indicated concentrations (n = 3, experimental triplicate).

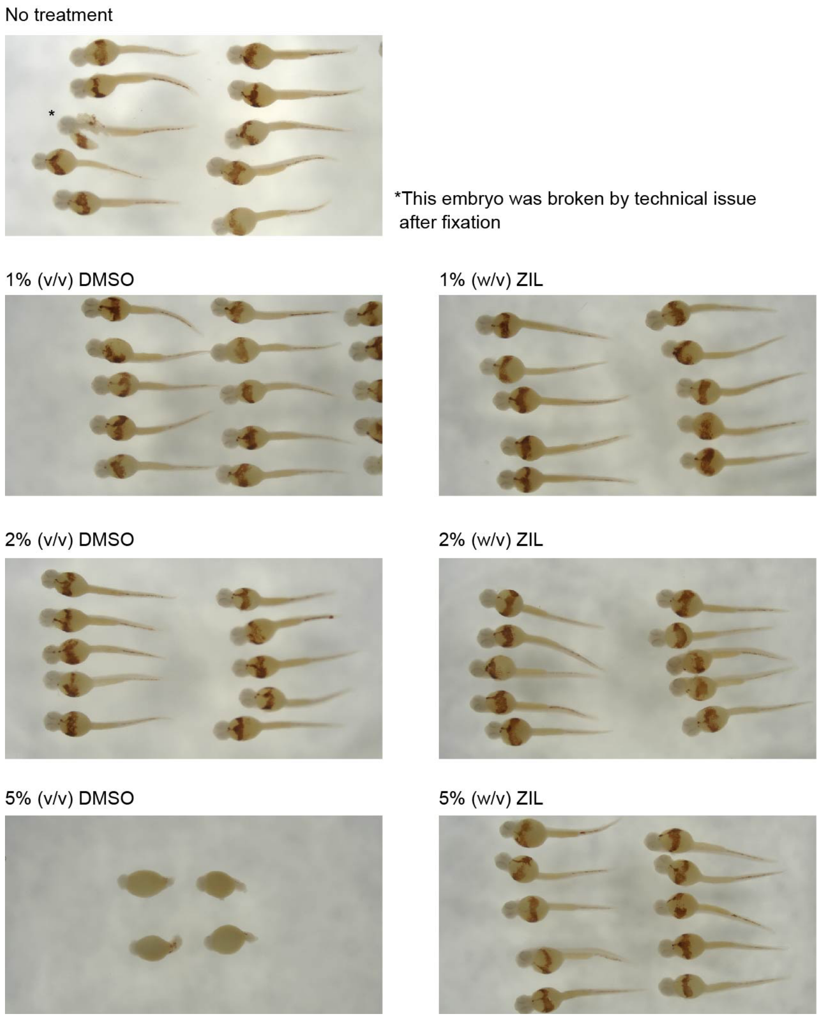

Supplementary Fig. 5 | ZIL does not induce malformation of zebrafish embryos at 48 hpf unlike DMSO. Regarding 5% (v/v) DMSO-treated samples, only four of 27 embryos survived at 48 hpf but with severe malformation. The embryos were stained with *o*-dianisidine to check erythropoiesis.

a

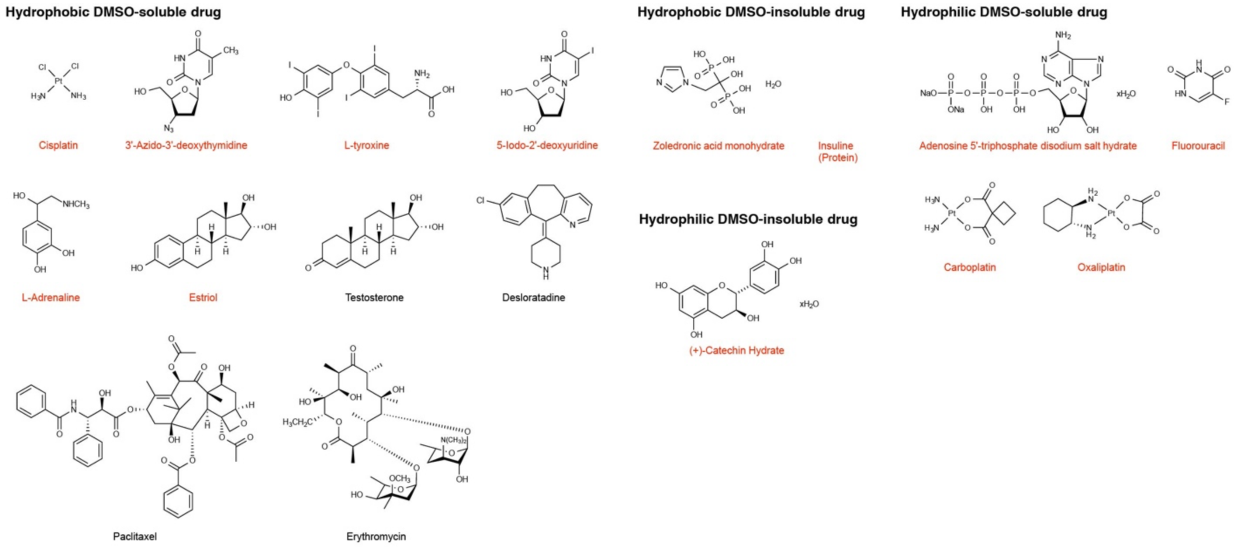

b

	Annotations	ZIL (wt%) in aqueous solution				Water	DMSO
		100	50	25	5		
**Hydrophobic DMSO-soluble drug**							
Cisplatin	Anticancer drug	soluble	soluble	soluble	soluble	insol.	soluble
3'-Azido-3'-deoxythymidine	Antiviral, Anti-HIV drug	soluble	soluble	soluble	soluble	insol.	soluble
L-thyroxine	Thyroid hormone	soluble	soluble	insol.	insol.	insol.	soluble
5-Iodo-2'-deoxyuridine	Antiviral	soluble	insol.	soluble	insol.	insol.	soluble
L-Adrenaline	Asthma agent	soluble	insol.	soluble	insol.	insol.	soluble
Estriol	Female hormone	soluble	insol.	insol.	insol.	insol.	soluble
Testosterone	Male hormone	insol.	insol.	insol.	insol.	insol.	soluble
Desloratadine	Antihistamine agent	insol.	insol.	insol.	insol.	insol.	soluble
Paclitaxel	Anticancer drug	insol.	insol.	insol.	insol.	insol.	soluble
Erythromycin	Antibiotic	insol.	insol.	insol.	insol.	insol.	soluble
**Hydrophobic DMSO-insoluble drug**							
Zoledronic acid monohydrate	Osteoporosis drug	soluble	soluble	soluble	soluble	insol.	insol.
Insulin	Diabetes drug	insol.	soluble	insol.	insol.	insol.	insol.
**Hydrophilic DMSO-soluble drug**							
Carboplatin	Anticancer drug	insol.	soluble	soluble	soluble	soluble	soluble
Oxaliplatin	Anticancer drug	soluble	soluble	soluble	insol.	soluble	soluble
(+)-Catechin Hydrate	Antioxidant	insol.	soluble	soluble	soluble	soluble	soluble
Fluorouracil	Anticancer drug	soluble	soluble	soluble	soluble	soluble	soluble
**Hydrophilic DMSO-insoluble drug**							
Adenosine 3'-phosphate	Drug for cardiac arrest	insol.	soluble	soluble	soluble	soluble	insol.

Supplementary Fig. 6 | ZIL and ZIL aq. dissolve various kinds of drugs. a, The structures of drugs. The drug named with red are soluble in ZIL and/or ZIL aq. **b**, The solubilities of the drugs in the indicated solvents.

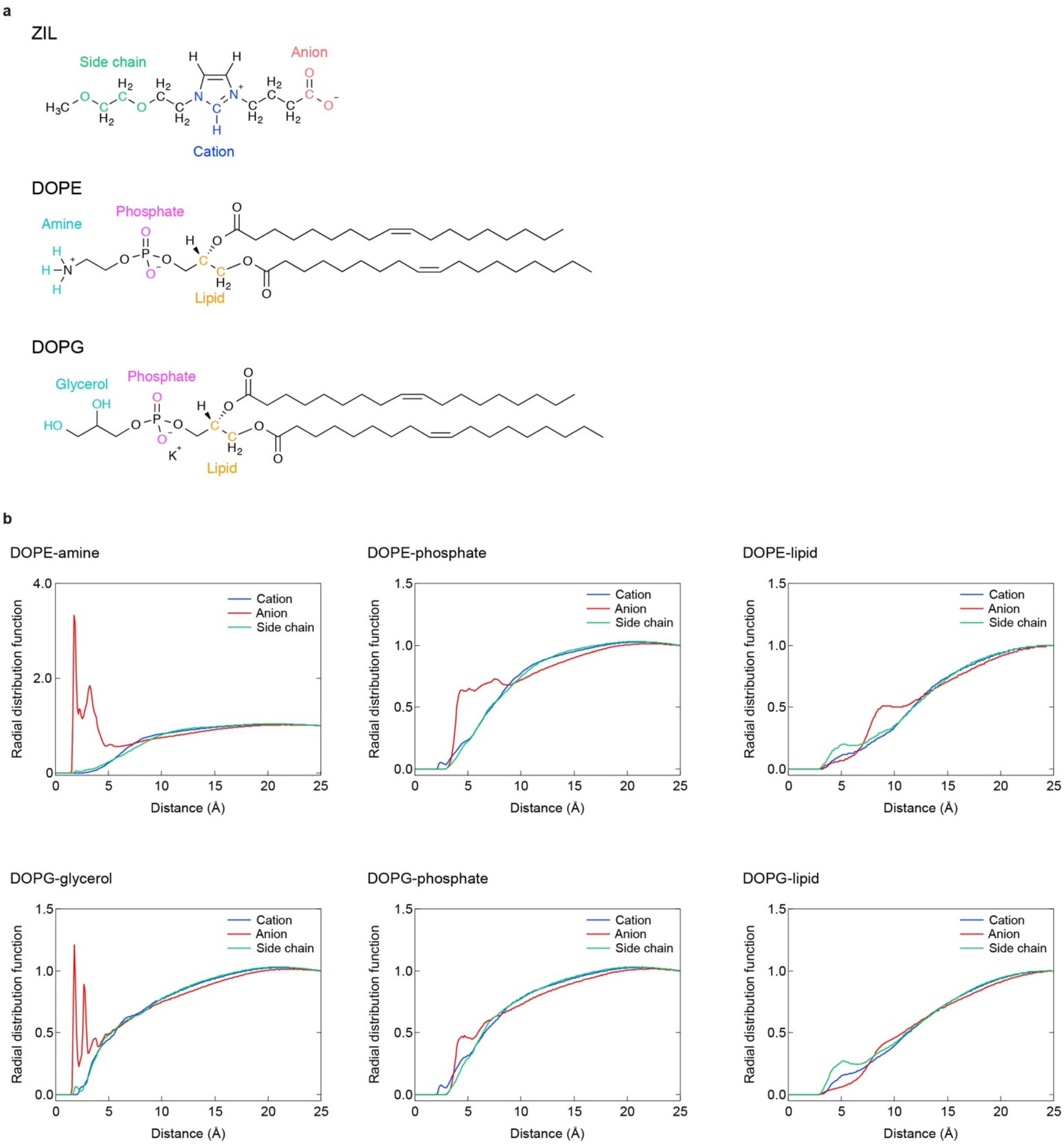

Supplementary Fig. 7 | ZIL molecules are concentrated at the surface of cells by electrostatic interaction between carboxylate of ZIL and amine of cell membrane. a, The atoms of ZIL, DOPE, and DOPG used in the calculation. The atoms used for the calculation were coloured. **b**, Radial distribution functions from the indicated part of lipids to cation, anion, and side chain of ZIL at 37 °C. DOPE: dioleoylphosphatidylethanolamine, DOPG: dioleoylphosphatidylglycerol.

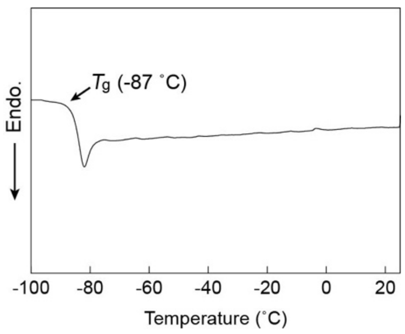

Supplementary Fig. 8 | ZIL aqueous solution (63 wt%) showed the glass transition at –87 °C. DSC measurement was conducted with the following ratio: cooling –1 °C/min, heating 5 °C/min. A weak glass transition signal was shown at –85 °C when measuring 5% (w/v) ZIL solution. The ratio of frozen water (free water) and glass water (bound water) in this case was roughly calculated from the heat of melting at around 0 °C. The heat of melting of ZIL solution at around 0 °C was –216 J/g and it is 92% of that of pure water (–235 J/g). Therefore around 8 wt% of the solution (5 wt% ZIL and 3 wt% water) turned to glass state. Here the 63 wt% ZIL solution (ZIL:water = 5:3 in weight) was subjected to DSC measurement, and a large glass transition signal was observed at –87 °C and no melting signal was observed.

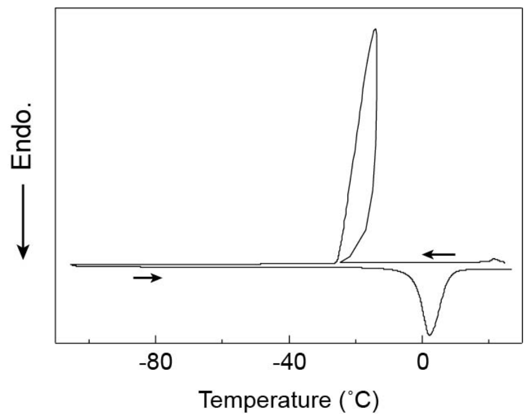

Supplementary Fig. 9 | Complete DSC chart of 5% (w/v) ZIL aqueous solution. (cooling – 1 °C/min, heating 5 °C/min). The exothermic signal in the chart seems irregular, but the phenomenon is due to great exothermic ice formation and reproducible.

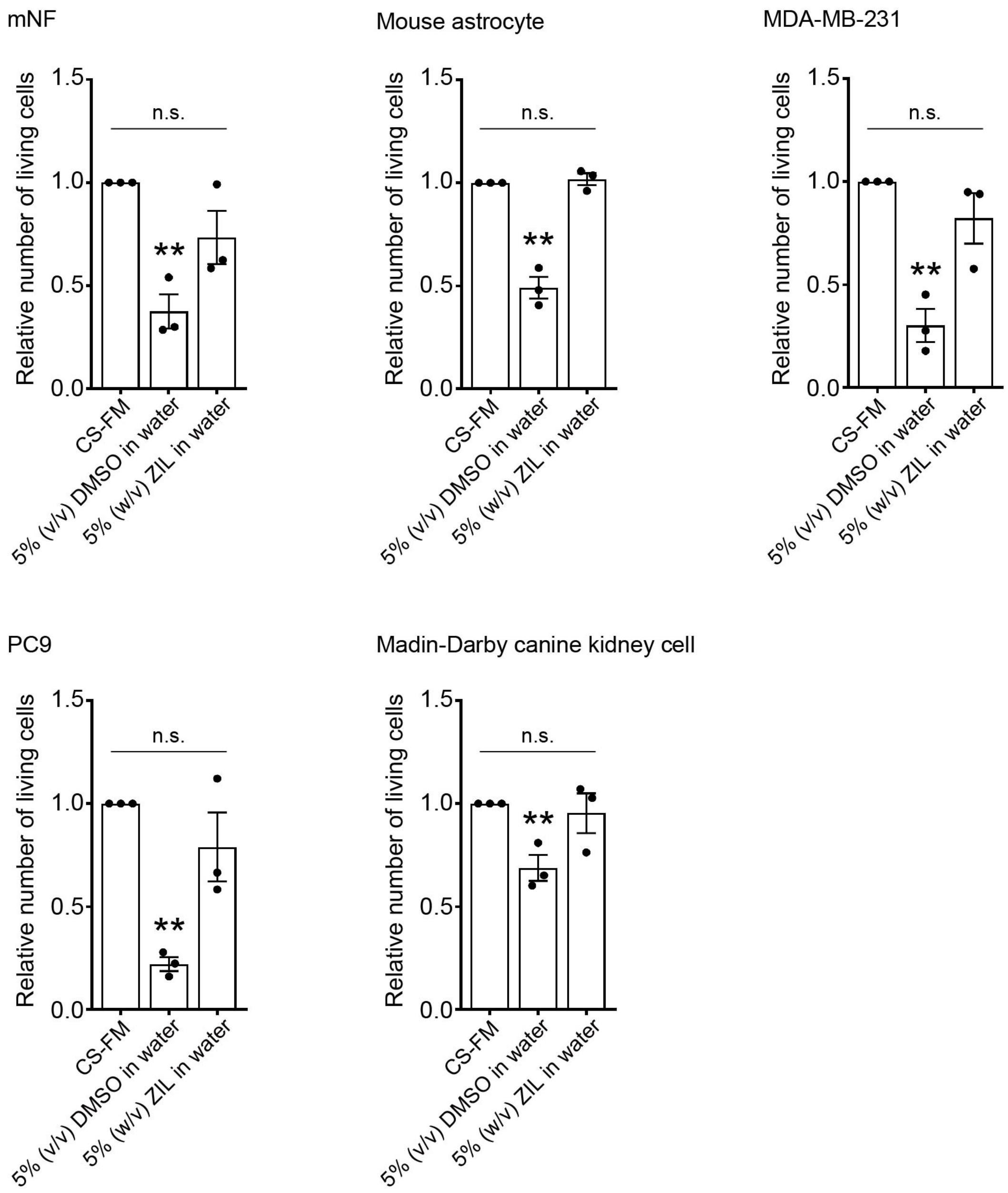

Supplementary Fig. 10 | 5% (w/v) ZIL aq. well works as a freezing medium for various types of cells. Relative number of living cells freeze-stocked at –85 °C for 3–5 days in the indicated freezing media (n = 3, biologically independent samples). All error bars indicate standard error.

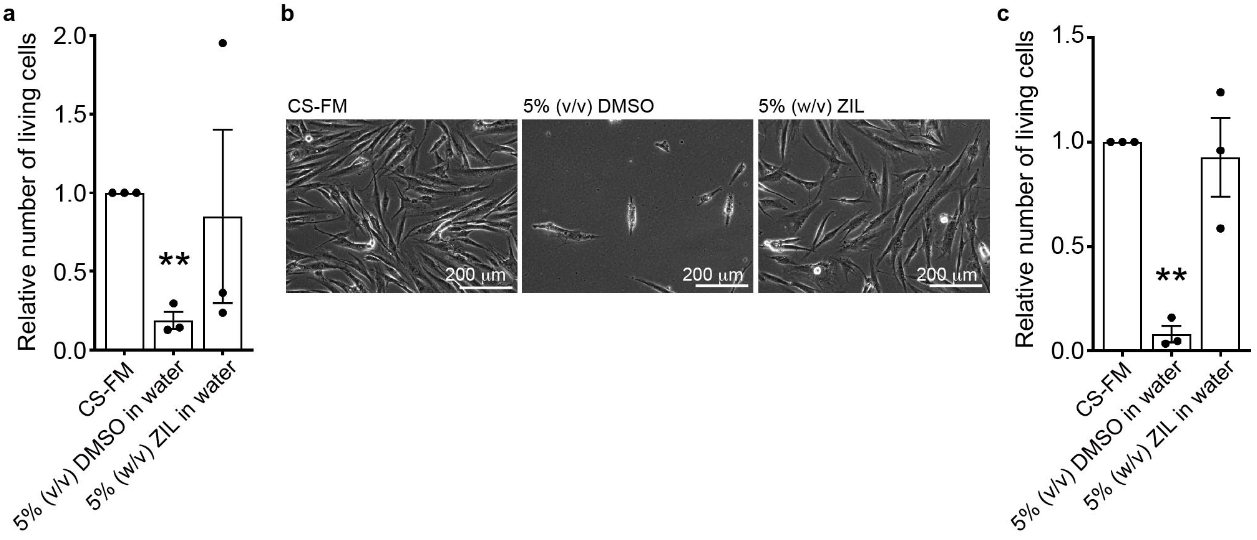

Supplementary Fig. 11 | 5% (w/v) ZIL aq. cryopreserved hNF-2 for one year. a, Relative number of living hNF-2 cells freeze-stocked at –85 °C for one year in the indicated freezing media (n = 3, biologically independent samples). **b**, **c**, Microscopic evaluation and relative number of living hNF-2 cells 24 h after recovery from one year-freezing (n = 3, biologically independent samples). All error bars indicate standard error. 5% (w/v) ZIL aq. successfully cryopreserved hNF-2 for one year with a similar efficacy given by the commercial cryoprotectant. Here, the absolute cell recovery with the commercial cryoprotectant was around 40%. It is natural in the cases of long-term cryopreservation because ‑85 °C is not enough for complete stop of cell metabolisms.

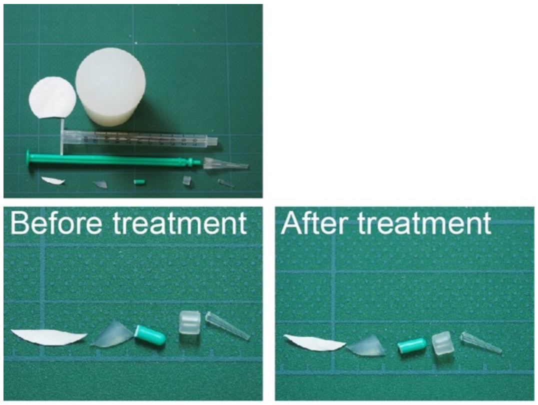

Supplementary Fig. 12 | ZIL is not corrosive to most plastic tools for biological experiments. (top) A picture of micro tip (polypropylene), syringe (outside and inside parts; polypropylene), silicone rubber stopper, and filter (PTFE) from right to left. (bottom) Pictures of the tools (left) before and (right) after stirring for 1 h in 100% ZIL at 80 °C. ZIL is a polar liquid and does not dissolve non-polar polymers such as polypropylene, polyethylene, polytetrafluoroethylene (PTFE), typical rubbers, and silicone rubbers, to the best of our knowledge. We here confirmed the durability of some plastics. Even after 1h stirring at 80 °C, there was no visible change for micro tip (polypropylene), syringe (polypropylene), silicone rubber stopper, and filter (PTFE). On the other hand, ZIL dissolves polar polymers and thus is not applicable to cellulose and polyvinyl alcohol. Cellulose filter is applicable when ZIL is aqueous solution (water content >20 wt%).

References

1. Lim, G. S., Zidar, J., Cheong, D. W., Jaenicke, S. & Klahn, M. Impact of ionic liquids in aqueous solution on bacterial plasma membranes studied with molecular dynamics simulations. *J. Phys. Chem. B* **118**, 10444-10459 (2014).
2. Kuroda, K. *et al.* Design of wall-destructive but membrane-compatible solvents. *J. Am. Chem. Soc.* **139**, 16052-16055 (2017).
