# Supplementary material for: Non-aqueous, zwitterionic solvent as an alternative for dimethyl sulfoxide in the life sciences
Source: Commun Chem. 2020 Nov 11;3:163. doi: 10.1038/s42004-020-00409-7 (PMC9814479; doi:10.1038/s42004-020-00409-7)
Supplement: Supplementary file 3 — Description of Additional Supplementary Files [file 42004_2020_409_MOESM3_ESM.pdf]

Description of Additional Supplementary Files

File Name: Supplementary Movie 1
Description: MD simulation of cell membrane in 5 wt% ZIL aqueous solution. The pictures were taken every 10 ns, from 0.5–1.5 μs. Water molecules are omitted for clarity.
